# Interspecific differences in foliar 1 PAHs load between Scots pine, birch, and wild rosemary from three polish peat bogs

**DOI:** 10.1007/s10661-016-5465-2

**Published:** 2016-07-08

**Authors:** Monika Mętrak, Ekonomiuk Aneta, Bogusław Wiłkomirski, Tomasz Staszewski, Małgorzata Suska-Malawska

**Affiliations:** 1 Faculty of Biology, Biological and Chemical Research Centre, University of Warsaw, ul. Żwirki i Wigury 101, 02-089 Warsaw, Poland; 2 Department of Environmental Protection and Modeling, The Jan Kochanowski University, in Kielce. Świętokrzyska 15, 25-406 Kielce, Poland; 3 Institute for Ecology of Industrial Areas, Kossutha 6, 40-844 Katowice, Poland

**Keywords:** PAHs, Bioindicator, Scots pine, Peat bog

## Abstract

Pine needles are one of the most commonly used bioindicators of polycyclic aromatic hydrocarbons (PAHs) in the environment. Therefore, the main objective of the current research was the assessment of PAHs accumulation potential of Scots pine (*Pinus sylvestris* L.) needles in comparison to wild rosemary (*Rhododendron tomentosum* Harmaja) and birch (*Betula* spp.) leaves. Our study was carried out on three peat bogs subjected to different degree of anthropopression, which gave us also the opportunity to identify local emission sources. Pine needles had the lowest accumulation potential from all the studied species. The highest accumulation potential, and hence carcinogenic potential, was observed for wild rosemary leaves. As far as emission sources are concerned, the most pronounced influence on atmospheric PAHs loads had traditional charcoal production, resulting in great influx of heavy PAHs. Observed seasonal changes in PAHs concentrations followed the pattern of winter increase, caused mainly by heating season, and summer decrease, caused mainly by volatilization of light PAHs.

**Electronic supplementary material:**

The online version of this article (doi:10.1007/s10661-016-5465-2) contains supplementary material, which is available to authorized users.

## Introduction

Polycyclic aromatic hydrocarbons (PAHs) are persistent organic pollutants (POPs) that occur ubiquitously in the environment. They are emitted by a broad range of natural and anthropogenic sources, mostly due to processes of incomplete combustion. Though there are considerable differences in the physicochemical properties of these compounds, they are all classified as semi-volatile organic compounds (SOCs), composed of two or more benzene rings, occurring both in the solid and gas phase. In comparison to heavier PAHs, lighter compounds are more volatile and more water-soluble. These differences have a decisive influence on PAHs emission, transportation, and deposition pathways (Mętrak et al. 2015; Oishi 2013; Satya et al. 2012; De Nicola et al. 2011; Ratola et al. 2011; Wiłkomirski et al. 2011; Lisowska 2010; Lehndorff and Schwark 2009).

Since PAHs show toxic properties for both plants and animals (including humans), great concern has been focused on them over the last years. Compounds from this group may have carcinogenic, mutagenic, teratogenic, and immunosuppressive influence on living organisms. Therefore, from among above 200 well-known PAHs, 16 compounds were listed by the US Environmental Protection Agency as priority pollutants and the European Union established a health-based standard for PAHs in air equal to 1 ng/m^3^. According to the EU directives, concentrations of PAHs in the air should be constantly monitored (Oishi 2013; Satya et al. 2012; De Nicola et al. 2011; Lisowska 2010; Sun et al. 2010; Malawska and Ekonomiuk 2008; EU Directive 2004/107/EC and Regulation 219/2009).

For biomonitoring purposes, vegetation has been widely used, since concentrations of contaminants in plant tissues can represent the integrated levels of these compounds in surrounding air (Simonich and Hites 1995). Though there are some difficulties in establishing the clear-cut quantitative relation between air and leaf concentrations of contaminants, plants have been used for determination of local and global contamination patterns, identification of pollution sources, or for assessment of seasonal changes in concentrations of contaminants (Oishi 2013; Sun et al. 2010). Amount of PAHs accumulated by plants depends on different factors, including type, size, and frequency of pollution influxes, exposure time, and morphological and physiological characteristics of the studied species (Oishi 2013; Li et al. 2010; Malawska and Ekonomiuk 2008; Malawska et al. 2006; Pal et al. 2002).

Plants can either uptake PAHs directly in vapor-phase via stomata or via outer cuticular lamellae; or accumulate them on the leaf surface, where they are subsequently incorporated into the lipid-rich cuticle layer (Piccardo et al. 2005; Lehndorff and Schwark 2004; Howsam et al. 2000; Franzaring 1997). Main constituent of cuticle is cutin–water-insoluble polymer of cross-linked hydroxy- and hydroxyepoxy fatty acids. On the outside, cutin is covered with a layer of epicuticular waxes, characterized by diversified structure, from amorphic to crystalline. Epicuticular waxes are a mixture of long-chained aliphatic and cyclic compounds such as alcohols, hydrocarbons, esters, fatty acids, and triterpenoids. Inside the cutin polymer, there are intracuticular waxes, which remain poorly characterized. There are also non-lipid components of cutin, i.a., polysaccharides and phenols (Li et al. 2010; Pallardy 2008; Kunst and Samuels 2003; Pal et al. 2002). Chemical characteristics of cutin combined with leaf structural features (shape, surface, presence and abundance of trichomes, glands, etc.) determine the amount of organic pollutants that can be potentially absorbed from the atmosphere (estimated per leaf dry mass or leaf area). As these features are species-dependent, there are several plants that are preferentially used as bioindicators of airborne contaminants, with general agreement that the better developed and preserved cuticle is, the more PAHs can be absorbed (Pallardy 2008; Piccardo et al. 2005; Pal et al. 2002). However, accumulation potential can be modified by interspecific and/or seasonal differences in proportions of cutin constituents (Ratola et al. 2011; Li et al. 2010; Pallardy 2008).

Though concentrations of PAHs accumulated by various plant species differ accordingly to morphological and physiological characteristics of their leaves, pollution profiles depend on sources, their distance from the area in question and on environmental conditions (Oishi 2013; Malawska and Ekonomiuk, 2008; Jouraeva et al. 2002; Malawska et al. 2002; Howsam et al. 2000). Therefore, we may assume, that PAHs profiles of different plant species growing in the same area will be comparable (same sources), yet concentrations of accumulated PAHs will differ depending on thickness and chemical composition of leaf cuticle. If pollution profiles differ between species growing in the same area, it is probably a result of qualitative profile modifications due to life form (canopy/understorey) and/or crown and leaf characteristics, typical for certain species.

In our study, we assessed PAHs pollution profiles of Scots pine (*Pinus sylvestris* L.), wild rosemary (*Rhododendron tomentosum* Harmaja), and birch (*Betula* spp.) growing in three peat bogs subjected to different intensity of anthropopression and influenced by different pollution sources. Knowing morphological and physiological differences between these species, we expected significant quantitative differences in accumulated PAHs. Yet, we were looking for qualitative differences in pollution profiles of studied species, in order to check if the proportions of absorption and accumulation are modified by species-dependent features. Moreover, we assumed, that PAHs ratios used by many authors (Yunker et al. 2011; Tobiszewski and Namiesnik 2012; Liu et al. 2015; Stogiannis and Lane 2016) will allow us to identify sources of PAHs for the studied peat bogs.

## Materials and Methods

### Study area

**Fig. 1:**
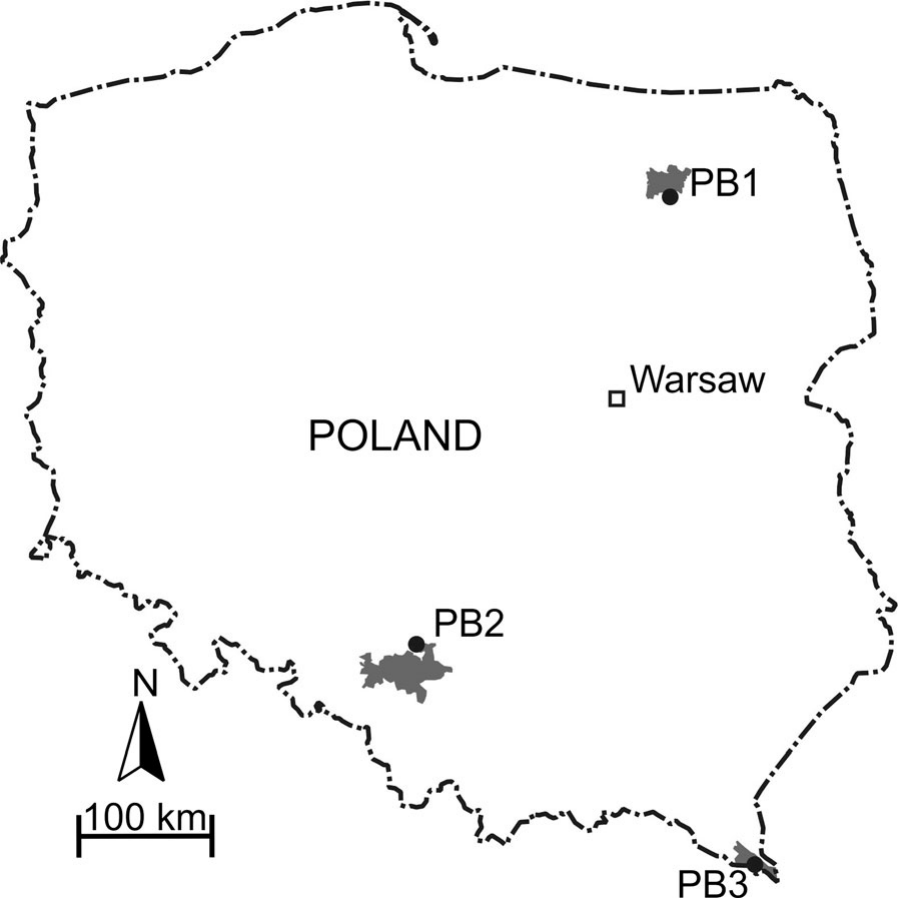
Location of the studied peat bogs. PB1 (53° 36.560′ N; 21° 37.832′ E), *gray area* depicts borders of the Masurian Landscape Park; PB2 (50° 31.401′ N; 19° 02.951′ E), *gray area* depicts borders of the Upper Silesian Industrial Region; PB3 (49° 04ʹ 46.98″ N; 22° 39ʹ 45.90″ E), *gray area* depicts borders of the Bieszczady National Park

For the purpose of our research, three forest bogs were chosen, all of them with visible hummocks and hollows. In each case, forest floor was densely covered with several species of *Sphagnum* moss, tussocks of cotton grass (*Eriophorum vaginatum* L.), and small shrubs from the Ericaceae family dominating on hummocks. The most common species of canopy layer were Scots pines with admixture of birches. Though covered by similar vegetation, chosen peat bogs were subjected to different intensity of anthropopression. Peat bog 1 (PB1, Fig. 1) is a main part of a wetland complex (with a fen and a transitional bog on its margins) developed via paludification in a postglacial area of the Masurian Lakeland (NE Poland). This part of Poland is an agricultural and tourist area, free of main industrial and urban PAHs sources. Described wetland complex belongs to the Masurian Landscape Park and is surrounded by the Puszcza Piska coniferous forest. Peat bog 2 (PB2, Fig. 1) is located in the Upper Silesia region (SW Poland) and originated from a lake established on fluvial sediments. Upper Silesia region has been heavily industrialized since the nineteenth century. Today, it is an area with enormous concentration of industry, called the Upper Silesian Industrial Region. In the vicinity of the PB2, there is an operating steel mill in Miasteczko Śląskie and an international airport in Pyrzowice. PB2 is surrounded by mainly coniferous forest of Lasy Lublinieckie. It is a NATURA 2000 site, supporting rare species of plants and animals. Peat bog 3 (PB3, Fig. 1) is located in the Bieszczady National Park (SE Poland) and was formed via paludification on an impermeable layer of Carpathian Flysch located in a valley of River Wołosatka. Though this is a sparsely populated, non-industrial region, traditional production of charcoal is continued there, resulting in huge influxes of organic pollutants. In 2012, there were 128 kilns working in the Bieszczady National Park, half of them in the vicinity of peat bog 3 (Marszałek and Kusiak 2013).

### Studied species

Pine (*Pinus* sp.) is one of the most commonly used bioindicators of airborne pollutants. Firstly, due to worldwide distribution of pine trees combined with relatively easy to establish age of their needles. Secondly, because of their leaf morphology, especially thick cuticle, comprising mostly polysaccharides and non-saponifiable biopolymers (cutan), with addition of cutin and waxes (Li et al. 2010). Moreover, they are rich in essential oils and resins (Pallardy 2008). Such chemical composition, combined with shape of leaves and tree crown, leads to effective scavenging and accumulation of semi-volatile organic compounds (Fernandez-Varela et al. 2015; Oishi 2013; Ratola et al. 2012; Amigo et al. 2011; Li et al. 2010; Malawska and Ekonomiuk 2008). For our study, we chose pine species common in eastern and central Europe—*P. sylvestris* L.

Birch (*Betula* sp.) is considered the most complex genus of all circumpolar genera, due to its frequent hybridization and high morphological variability (Orav et al. 2011). As separation between *Betula pendula* Roth and *Betula pubescens* Ehrh. is usually problematic, we decided to classify our samples as *Betula* spp., potentially comprising leaves of *B. pendula* and *B. pubescens*. And their hybrid *Betula* × *aschersoniana* Hayek. Leaves of birch species are usually covered by glands producing resins, which are a mixture of phenolics and triterpenoids. Glands are highly metabolically active especially in young leaves. In case of *B. pubescens*, leaves are additionally covered by hairs. The main constituents of birch leaves are flavonoids, including epicuticular flavonoid aglycones suspended in resinous, sticky matrix covering surface of leaves (Orav et al. 2011; Valkama et al. 2003). Furthermore, periplasmic spaces in birch leaves are often filled with lipid droplets or myelin-like material (Valkama et al. 2003). Rich in lipophilic compounds, birches may be viewed as a valuable alternative to pines, as far as bioindication in the summer season is concerned.

Wild rosemary (*R. tomentosum* Harmaja) is an evergreen shrub, which is common on Polish plains and is present on dispersed sites in Polish uplands and mountains. This species, under a partial protection in Poland, is typical for raised and transitional bogs, and for coniferous swamp forests (Kłosowski and Kłosowski 2006). The upper leaf surface of wild rosemary is shiny and glabrous, while the lower surface is densely covered with rufous hairs (Dampc and Luczkiewicz 2013). Wild rosemary plants are rich in lipophilic compounds, such as flavonoids, phenolics, and coumarins. Moreover, they produce huge amounts of essential oils, comprising at least 90 different chemicals. Therefore, these species is traditionally used in folk medicine and as an insect repellent. Currently, it is investigated for bioactive compounds showing antimicrobial, antioxidant, and antidiabetic properties (Dampc and Luczkiewicz 2013).

### Sampling

Plant material was collected in July and October 2002 and 2003, and in case of pine, additionally in January 2003. Each time, from the central part of every peat bog one composite sample was taken comprising leaves/needles of 5 to 10 random individuals from each species. In case of *R. tomentosum*, to avoid the effect of canopy on deposition of pollutants, only plants growing in the openings were sampled. Plant samples were wrapped in aluminum foil and transported to the laboratory at ca. 4 °C. Before analyses, they were stored at −20 °C. Extraction procedure was carried out on defrosted plants.

### Chemical analyses

The following 17 PAHs were determined in the collected plant material: acenaphthylene, acenaphthene, fluorene, phenanthrene, anthracene, fluoranthene, pyrene, benzo[a]anthracene, chrysene, benzo[b]fluoranthene, benzo[k]fluoranthene, benzo[e]pyrene, benzo[a]pyrene, perylene, indeno[123-cd]pyrene, dibenzo[ah]anthracene, and benzo[ghi]perylene. The extraction of plant samples was performed with the use of dichloromethane. Further purification was carried out on Florisil. The PAHs content analysis was performed using a gas chromatograph equipped with a mass selective detector GC/MSD HP and a non-polar capillary column HP. The details of chromatographic parameters were described in (Malawska and Ekonomiuk 2008). The procedures described earlier were checked for recoveries and reproducibility. Prior to extraction, recoveries were investigated by spiking *P. sylvestris*, *Betula* spp., and *R. tomentosum* leaves with four increasing amounts of standards. For the sum of compounds analyzed, recovery results varied between 81 and 96 %. Reproducibility was calculated on replicate analyses, giving an error between 3.1 and 8.4 %. Every tenth sample was extracted and analyzed in duplicate. After an analysis of each tenth sample, standard samples with known PAHs content were analyzed. Analyses of blanks were performed for every eight samples. PAHs concentration in all blank values was below the detection limit. Mean recovery rates for individual PAHs are shown in the Appendix (Table 1). Analyses of PAHs were carried out in the Central Chemical Laboratory of Polish Geological Institute.

### Carcinogenic potential

Carcinogenic potential (CP) of the PAH deposit in the studied samples was calculated according to the following formula:

$$ CP=\frac{{\displaystyle \sum \left(\mathrm{amount}\ \mathrm{of}\ \mathrm{carcinogenic}\ PAH\times \%\mathrm{of}\ \mathrm{its}\ \mathrm{carcinogenic}\ \mathrm{properties}\ vs\ BaP\right)}}{100} $$

with the use of estimations of carcinogenic properties provided by the EPA Tasmania (2012).

### Statistical analyses

As studied parameters failed the assumptions of parametric tests (normal distribution and/or equal variances), non-parametric tests were used. In order to compare pollution profiles of the three studied locations, Friedman ANOVA for dependent samples was performed (samples from the same species and from the same month were compared between the bogs). Similar analysis was performed to compare pollution profiles of the three studied species (samples from the same place and from the same month were compared between the species). Since perylene originates mostly in bacterial and geological processes, it was excluded from statistical analyses.

## Results

### Pollution profiles of the studied peat bogs

**Fig. 2:**
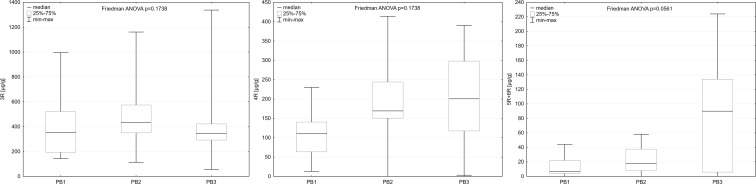
Comparison of 3-, 4-, and 5 + 6-ring PAH concentrations between the studied peat bogs. Due to great differences in concentrations of PAHs from these groups, different ranges of *Y*-axis were used

In the samples collected from all three bogs dominated light, 3- and 4-ring PAHs, with the mean concentrations of phenanthrene of more than 300 μg/g for all locations, followed by fluoranthene reaching on average almost 100 μg/g. Mean concentrations of pyrene, fluoranthene and acenaphthylene for the studied locations ranged from 20 to 50 μg/g. In case of 3-ring PAHs, there were no statistically significant differences observed between the bogs (Fig. 2), with the exception of acenaphthene concentration (*p* value in Friedman ANOVA 0.0153), which was the lowest in the samples from PB1 (mean value 2.6 μg/g, mean value for PB2 4.7 and for PB3 3.9). The lightest PAHs constituted from 66 to 92 % of total PAHs in the samples from PB1, from 58 to 100 % in the samples from PB2, and from 44 to 96 % in samples from PB3. Observed differences were statistically significant with *p* value in Friedman ANOVA equal 0.0065.

In case of 4-ring PAHs, observed differences in their concentrations were statistically significant only for chrysene (*p* value in Friedman ANOVA 0.0036) and followed the pattern PB1 < PB2 < PB3, with the mean values reaching 10.2, 19.0, and 32.3 μg/g consecutively. Percentage participation of 4-ring PAHs in total PAHs ranged from 8 to 31 % in the samples from PB1, from 0 to 39 % in the samples from PB2, and from 4 to 37 % in the samples from PB3. As in the case of 3-ring PAHs, these differences were statistically significant (*p* value in Friedman ANOVA 0.0438).

Concentrations of the heaviest PAHs (5 and 6 rings) varied significantly between the studied bogs and followed the pattern PB1 < PB2 < PB3 (*p* value for benzo[k]fluoranthene = 0.0014, *p* value for benzo[e]pyrene = 0.0161, *p* value for benzo[a]pyrene = 0.0423, *p* value for indeno[123-cd]pyrene = 0.0076, *p* value for benzo[ghi]perylene = 0.0014). The same trend was observed for benzo[b]fluoranthene with *p* value in Friedman ANOVA reaching 0.0617. Concentrations of heavy PAHs in samples from PB3 were up to 10 times higher than in samples from PB1 and PB2. Results for dibenzo[ah]anthracene are highly biased, as this compound was recorded only in a few samples, which made proper comparisons impossible. Yet, it is worthy to notice that dibenzo[ah]anthracene was found only in the samples from PB2 and PB3. The heaviest PAHs constituted from 0 to 8 % of total PAHs in the samples from PB1, from 0 to 9 % in the samples from PB2 and from 0 to 22 % in the samples from PB3 (*p* value in Friedman ANOVA 0.0757). Thus, samples from PB3 were significantly poorer in light PAHs and enriched in heavy PAHs (4-, 5-, and 6-ring PAHs).

Detailed information on PAHs’ concentrations and statistical significance of observed differences are presented in Supplementary materials in Tables 2, 3, and 4.

As far as PAH ratios are concerned, observed differences had no statistical importance, yet some trends were clearly visible (Table 1). Anth/(Anth + Phen) ratio of the samples was typically far below 0.1, with the exception of a few samples from PB3, in which it exceeded 0.20 (*p* value in Friedman ANOVA 0.0755). Fluo/(Fluo + Pyr) ratio of the samples ranged between 0.22 and 1.00, with slightly lower values observed in the samples from PB3 (*p* value in Friedman ANOVA 0.0755). Bzaan/(Bzaan + Chry) ratio of the samples ranged between beyond detection limit to 0.36 and was very similar for the samples from all three peat bogs (*p* value in Friedman ANOVA 0.1146). Results for Ind/(Ind + Bzper) ratio should be interpreted with caution, hence these compounds occurred simultaneously only in a few samples (most frequently in the samples from PB3), which made proper comparisons impossible.

**Table 1:** Comparison of PAHs ratios and carcinogenic potential (CP) in plant samples from three studied peat bogs

PAHs ratio/CP		PB1 (*N* = 12)	PB2 (*N* = 12)	PB3 (*N* = 12)	Friedman ANOVA *p* value
An/(Pn + An)	mean	0.02	0.03	0.06	0.0755*
	median	0.02	0.03	0.03	
	range	0.00–0.08	0.02–0.07	0.02–0.22	
Fl/(Fl + Py)	mean	0.52	0.52	0.46	0.0755*
	median	0.52	0.44	0.39	
	range	0.32–0.93	0.22–1.00	0.22–0.90	
BaA/(BaA + Ch)	mean	0.22	0.18	0.22	0.1146
	median	0.24	0.21	0.26	
	range	0.00–0.31	0.00–0.26	0.00–0.36	
IP/(IP + BgP)	mean	0.08	0.09	0.26	0.0760*
	median	0.00	0.00	0.36	
	range	0.00–1.00	0.00–0.55	0.00–0.52	
CP	mean	3.83	5.87	23.04	0.0254**
	median	2.50	5.99	21.82	
	range	0.00–12.11	0.00–18.32	0.00–61.56	

*p values <0.1; **p values <0.05; ***p values <0.001)

Samples from different bogs varied significantly regarding carcinogenic potential (CP), which was the highest for the samples from PB3 (median value 21.81; *p* value in Friedman ANOVA 0.0254). Detailed information on PAH ratios and carcinogenic potential is presented in Table 1.

### Pollution profiles of the studied plants

**Fig. 3:**
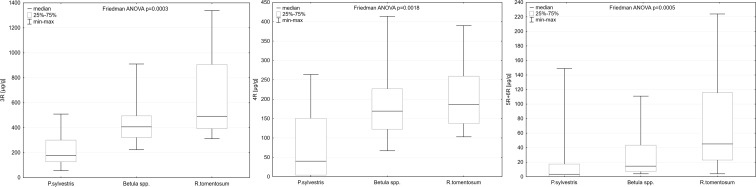
Comparison of 3-, 4-, and 5 + 6-ring PAHs concentrations between the studied species. Due to great differences in concentrations of PAHs from these groups, different ranges of *Y*-axis were used

Differences in PAHs concentrations between *P. sylvestris*, *Betula* spp., and *R. tomentosum* for almost all of the studied compounds were statistically significant, the only exceptions being two light PAHs: acenaphthylene (*p* value = 0.5875) and fluorene (*p* value = 0.0590) and two heavy PAHs: indeno[123-cd]pyrene (*p* value = 0.0863) and dibenzo[ah]anthracene (*p* value = 0.1353). For phenanthrene, fluoranthene, and benzo[k]fluoranthene, *p* values in Friedman ANOVA were below 0.001. For anthracene, chrysene, benzo[b]fluoranthene, benzo[e]pyrene, benzo[a]pyrene, and benzo[ghi]perylene, *p* values in Friedman ANOVA were below 0.01. For acenaphthene, pyrene, and benzo[a]anthracene, *p* values in Friedman ANOVA tests were below 0.05. The highest PAHs concentrations were recorded in the samples of *R. tomentosum* and the lowest in the samples of *P. sylvestris*. Exceptions from this general rule were observed only for the lightest PAHs, including acenaphthene, acenaphytlene and fluoranthene. Yet, they did not influence the observed pattern in concentrations of 3-, 4-, and 5 + 6-ring PAHs (Fig. 3). As dibenzo[ah]anthracene was recorded only in a few samples, mostly in *R. tomentosum*, proper comparisons in case of this compound were impossible.

The studied species did not significantly differ as far as PAH ratios are considered. However, huge differences were observed regarding carcinogenic potential (CP), which was three times higher in the samples of *R. tomentosum* than in the samples of *P. sylvestris* or *Betula* spp. Detailed information on concentrations of PAH ratios and CP index in the samples of different species is presented in Table 2.

**Table 2:** Comparison of PAHs ratios and carcinogenic potential (CP) in samples of *P. sylvestris*, *Betula* spp., and *R. tomentosum*

PAHs ratio/CP		*P. sylvestris* (*N* = 12)	*Betula* spp. (*N* = 12)	*R. tomentosum* (*N* = 12)	Friedman ANOVA *p* value
An/(Pn + An)	mean	0.07	0.03	0.02	0.7584
	median	0.03	0.02	0.02	
	range	0.01–0.22	0.00–0.04	0.00–0.05	
Fl/(Fl + Py)	mean	0.66	0.38	0.46	0.0970*
	median	0.62	0.36	0.46	
	range	0.26–1.00	0.22–0.58	0.22–0.75	
BaA/(BaA + Ch)	mean	0.16	0.21	0.26	0.3229
	median	0.20	0.21	0.26	
	range	0.00–0.36	0.14–0.31	0.20–0.31	
IP/(IP + BgP)	mean	0.08	0.16	0.19	0.4046
	median	0.00	0.00	0.00	
	range	0.00–0.52	0.00–1.00	0.00–0.54	
CP	mean	5.55	7.83	19.38	0.0002***
	median	0.62	5.85	11.05	
	range	0.00–38.71	0.60–25.83	0.80–61.56	

*p values <0.1; **p values <0.05; ***p values <0.001

### Seasonal changes in PAHs concentrations

**Fig. 4:**
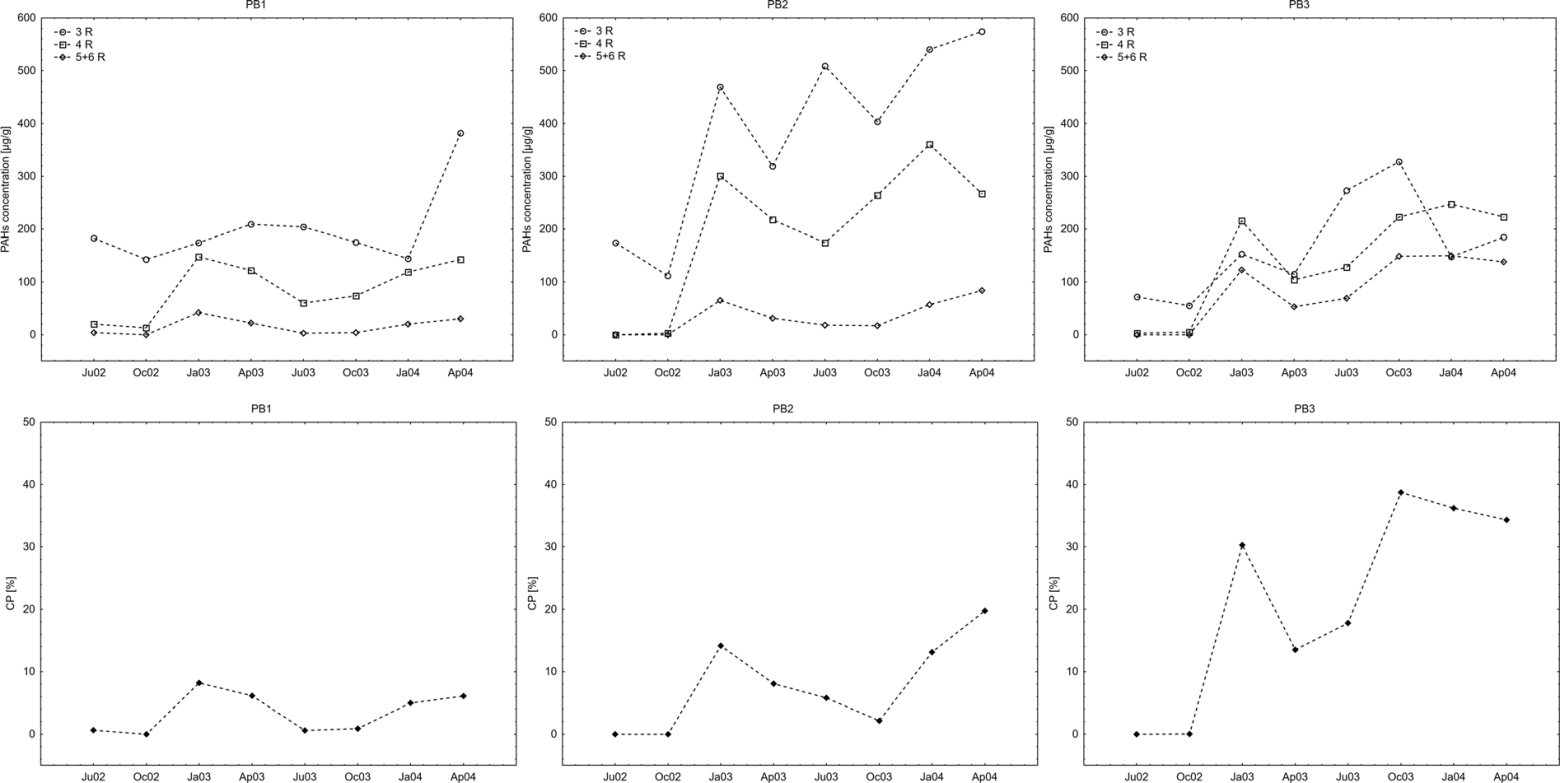
Seasonal changes in concentrations of 3-, 4-, and 5 + 6-ring PAHs in *P. sylvestris* needles (*upper row*) and in their carcinogenic potential (CP) (*lower row*)

Seasonal changes in concentrations of 3-, 4-, and 5 + 6-ring PAHs in *P. sylvestris* needles are shown on Fig. 4. In case of 4-ring PAHs, changes resulting from heating season were clearly visible, with noticeable increases in concentrations in October and January. The same pattern could be observed for 5 + 6-ring PAHs, yet the increases were not so well pronounced. Fluctuations in concentrations of light and mobile 3-ring PAHs did not fully reflect heating season.

Changes in carcinogenic potential (CP) of PAHs deposited in *P. sylvestris* needles followed the pattern of heavy PAHs, with noticeable increases during autumn and winter months (Fig. 4).

## Discussion

### PAHs composition and sources

In all of the studied samples, light 3- and 4-ring PAHs dominated, with phenanthrene having the highest overall concentrations. This is in line with predominant uptake of the lighter PAHs by plants instead of the heavier ones. Low molecular weight PAHs (two and three rings) exist in the atmosphere mostly in the gaseous phase, while the heavier compounds (five and six rings) are found in the particulate phase. Therefore, the lighter PAHs can directly penetrate the inner leaf compartments via stomata or outer cuticular lamellae, while the heavier ones, after being desorbed from the particles, are accumulated into the leaf waxy layer. As such, they are exposed to effects of water and photodegradation (Terzaghi et al. 2013; Ratola et al. 2011; 2012; Amigo et al. 2011; De Nicola et al. 2011; Lehndorf and Schwark 2004; Franzaring 1997). Thus, while using bioindicators of airborne organic pollutants, we should take into consideration general condition of leaf/needle surface, that is a result of combined effects of leaf age (age-related degradation of waxes, including transition from tubular into amorphous structure); health (presence of molds plays an important role in accumulation of atmospheric particles); and influence of environmental stress (erosion of waxes was observed as a result of atmospheric pollution, i.a., O_3_, SO_x_, NO_x_) (Terzaghi et al. 2013; Sæbø et al. 2012; De Nicola et al. 2011; Ratola et al. 2010; Riikonen et al. 2010; Piccardo et al. 2005; Jouraeva et al. 2002; Crossley and Fowler 1986).

Accumulation of PAHs in plant tissues reflects the composition of PAHs in surrounding air over time and can be used to assess regional and global contamination patterns and to identify point sources of pollution (Fernandez-Varela et al. 2015; Ratola et al. 2012; De Nicola et al. 2011; Sun et al. 2010; Malawska et al. 2003; Holoubek et al. 2000). According to the processes in which they are produced, PAHs can be divided into three categories: (1) mostly light compounds related to combustion of oil, coal, and their byproducts (petrogenic); (2) heavier compounds (4–6 rings) related to anthropogenic combustion processes and forest fires (pyrogenic); and (3) compounds linked to microbial or diagenetic processes, mostly perylene (biogenic) (Fernandez-Varela et al. 2015; De Nicola et al. 2011). In our case, petrogenic PAHs dominated and remained indiscriminative for our study sites, while the least-abundant heavy compounds (5–6 rings) were present in the highest concentrations in the samples from PB3, showing potential huge impact of pyrogenic sources. In order to identify emission sources for our study locations, we calculated PAH ratios according to Yunker et al. (2011). Surprisingly, the differences in these ratios between the studied locations turned out to be of no statistical importance. In case of An/(Pn + An) (*p* value 0.0755), mean values for all of the studied locations were below 0.1, which indicates combustion of petroleum as the major source of emissions. Values above 0.1 were recorded exclusively in a few samples from PB3, indicating influence of some mixed sources, including biomass combustion. For Fl/(Fl + Py) mean values for all three locations were about 0.5 (*p* value 0.0755), which is characteristic for mixed emission sources. Similarly, mean values for BaA/(BaA + Ch), which were below 0.35 for all the locations (*p* value 0.1146), are typical for mixed emission sources. In case of IP/(IP + BgP), values for PB1 and PB2 are below 0.2, which points to petroleum sources, while for PB3, values are between 0.2 and 0.5, which once again indicates mixed emission sources. Yet, this ratio is also of no statistical importance, as far, as differences between the locations are considered (*p* value 0.0760).

These slight shifts in PAH ratios indicating influences of mixed emission sources in the samples from PB3, together with statistically important differences in concentrations of heavy PAHs, can be explained by about 100 charcoal kilns working in the Bieszczady National Park, where PB3 is located. According to Lisowska (2010), the process of wood burning at a reduced amount of oxygen increases production of 4–6-ring PAH up to 80 %, while during coal-burning processes up to 90 % of light compounds are emitted.

Increased amount of heavy PAHs emitted, results also in high carcinogenic potential of emissions from charcoal production, which is reflected by five times higher CP index for samples from PB3 in comparison with samples from PB1 and PB2 (*p* value of 0.0254). Interestingly, in comparison to results presented in Malawska and Ekonomiuk (2008), overall concentrations of PAHs from PB3 are much lower, especially in case of heavy PAHs. This may be an effect of decrease in number of kilns operating in Bieszczady over the time between our sampling campaigns.

### Differences in PAHs accumulation in the studied plant species

Conifer species, especially Scots pine (*P. sylvestris*), are considered good bioindicators for atmospheric pollutants, due to their epicuticular waxy layer, wide distribution, and easy identification (Oishi 2013; Ratola et al. 2011; Sun eta al. 2010). However, as far as PAHs are concerned, they turned out to be the least efficient bioaccumulators of the species described in this paper. In comparison to birch (*Betula* spp.) and wild rosemary (*R. tomentosum*) leaves, pine needles accumulated the lowest amounts of all PAHs concerned in this study, with the exception of acenaphthylene, fluoranthene, dibenzo[ah]anthracene, and indeno[123-cd]perylene. Our previous observations confirm great accumulation potential of *R. tomentosum*. However, previously observed differences were less pronounced than presented in this article (Malawska and Ekonomiuk 2008; Malawska et al. 2002).

Though significant differences in amounts of PAHs were observed, the proportions of PAHs of different molecular weights were the same for all of the studied species. The highest content was recorded for light 3-ring PAHs, followed by 4- and 5 + 6-ring compounds. This pattern of PAHs incidence in plant samples is an effect of processes occurring in the atmosphere and on the leaf surface, influencing PAHs dependently on their physical and chemical features (Terzaghi et al. 2013; Amigo et al. 2011; De Nicola et al. 2011; Ratola et al. 2011; Lehndorf and Schwark 2004; Franzaring 1997). As such, it was also observed in our previous studies on Polish peat bogs (Malawska et al. 2002; Malawska and Ekonomiuk 2008).

Differences between plant species in accumulation of atmospheric pollutants can be caused by many factors of morphological and physiological origin. Plants are efficient scavengers of atmospheric particulate matter, with which they remove PM-associated contaminants. Trees are considered the most effective type of vegetation for this purpose, due to their large total leaf area (Terzaghi et al. 2013; Sæbø et al. 2012; Jouraeva et al. 2002). Interspecific variations in size and structure of tree crowns, and size and geometry of leaves lead to differences in air movements inside the canopy, resulting in fluctuations of PM deposition on leaves (Oishi 2013; Rodriguez et al. 2012; Sæbø et al. 2012). In this aspect, coniferous species are considered to be more effective than broadleaved species—the long and narrow leaves of coniferous species being more easily hit by particles in the air than large and flat leaves, which have thicker boundary layers (Sæbø et al. 2012; Beckett et al. 1998).

However, Terzaghi et al. (2013) observed that leaves of cornel and maple sampled at the urban background site (a small wood 150 m away from the nearest road), accumulated similar amounts of particles as 0-year needles of pine, which was located at the polluted urban site and therefore exposed to a higher air concentration of particulate matter. Furthermore, according to Sæbø et al. (2012), among the studied tree species, *B. pendula* was characterized by the highest accumulation of fine particulate matter (PM_0.2_) and this seemed to be related to the waxes on the leaves of this species. According to Paoletti et al. (2011), observed differences in scavenging potential between the species can be extremely large, e.g., 2.6 g PM_10_ per tree in the case of *Carpinus betulus*, compared to 164 and 182 g PM_10_ in case of *Pinus pinea* and *Aesculus hippocastanum*. Interestingly, high concentrations of particular matter were observed for low growing shrub species. Presumably, because they are more exposed to soil splash on the leaves than vertically growing trees (Sæbø et al. 2012; Dzierżanowski et al. 2011). This, apart from leaf features and high content of essential oils (Dampc and Luczkiewicz 2013), may explain high concentrations of PAHs recorded in *R. tomentosum* leaves during our study.

Additionally, capturing of PM can be enhanced by the presence of trichomes on leaves or by rough leaf surface in general (Ratola et al. 2012; Sæbø et al. 2012; Hwang et al. 2011). In our case, such structures were present on some leaves of *Betula* spp. and in vast amounts on leaves of *R. tomentosum*. As far as samples of birch are concerned, each composite sample may have included leaves with different area covered with trichomes—the largest in case of leaves of *B. pubescens*, medium in case of leaves of *Betula* × *aschersoniana* and the smallest in case of *B. pendula*. Scavenging of PM by plants can also be modified by other species-specific leaf characteristics, such as orientation, petiole length, and rigidity or wettability (Terzaghi et al. 2013). No matter the leaf characteristics, the dominant fraction of PM on leaf/needles is low-diameter PM_2.5_, with high specific surface area, which increases binding capacity for PAHs. PAHs adsorbed on the surface of PM_2.5_ can migrate into the cuticle and underlying leaf structure (Terzaghi et al. 2013).

Apart from shape- and surface-related features of leaves, leaf cuticle plays a crucial role in retention of particulate matter and sorption of PAHs. In this aspect, not only its chemical composition (e.g., fluctuations in concentrations of particular constituents responsible for hydrophobicity), but also physical structure of cuticle (e.g., thickness, morphology, alteration with age, presence of crystals) is important (Oishi 2013; Terzaghi et al. 2013; Li et al. 2010; Jouraeva et al. 2002). In our case, an unpublished pilot study showed that the amount of hexane extractable lipids is higher in birch leaves than in 0-year-old pine needles—for samples collected in spring 83 mg/g dry weight (d.w.) for birch and 35 mg/g d.w. for pine, and in autumn 69 mg/g d.w. for birch and 51 mg/g d.w. for pine. High amount of lipids, particularly of waxes, in birch leaves was confirmed by Sæbø et al. (2012), stating that *B. pendula* was rich in waxes and accumulated most of the PM in the waxes. However, the chemical composition of waxes is also significant and high amounts of lipids (including waxes) are not necessarily correlated with high amounts of contaminants deposited on the leaf (Sæbø et al. 2012; Li et al. 2010; Jouraeva et al. 2002). According to Li et al. (2010), the epicuticular waxy layer was an effective sorption medium for PAHs, yet it was not the main contributor for PAHs accumulation. The key contributor was identified as cutin, which stored more than 90 % of PAHs. The role of the second component of plant cuticle, cutan, in sorption of PAHs was insignificant due to the presence of sugar fractions, causing dispersal of cutan components among hydrophilic domains (Li et al. 2010). Therefore, though the potential sorption capacity of cuticle components increased with the increase of aromatic C content, their role in sorption was regulated by the amorphous cellulose component. With the presence of amorphous cellulose, sorption was limited to non-polar aliphatic moieties. Without the presence of amorphous cellulose, both aromatic and aliphatic moieties were effective in sorption (Li et al. 2010).

While studying accumulation of PAHs by different species, we should take into consideration modifications in structure and composition of leaf cuticle resulting from age and various environmental stresses to which plants may be subjected (Terzaghi et al. 2013; Sæbø et al. 2012; De Nicola et al. 2011; Ratola et al. 2010; Riikonen et al. 2010; Piccardo et al. 2005; Jouraeva et al. 2002; Crossley and Fowler 1986).

### Seasonal changes of PAHs concentration in pine needles

Concentrations of PAHs during the life of a needle are influenced by two contradictory processes—accumulation, causing increase of concentrations over time and accordingly to intensity of emissions, and on the other hand, processes leading to decrease in concentrations over time, such as photodegradation and resuspension into the atmosphere (in case of the light PAHs) or leaf cuticle degradation and weather effects (in case of the heavy PAHs). Usually, heavy PAHs bound to particulate matter and deposited on the leaf surface are more prone to degradative processes (Alves et al. 2012; Ratola et al. 2010).

Regarding the seasonal variation of PAHs concentrations in pine needles, we observed an increasing tendency in PAHs concentrations in autumn and winter and decreasing tendency from winter to summer. These trends were visible in the samples from all the three research sites. Such trends are climate dependent and are commonly observed in the Northern Hemisphere (Fernandez-Varela et al. 2015; Alves et al. 2012; Amigo et al. 2011; Ratola et al. 2010). Higher concentrations of PAHs in autumn and winter reflect a higher incidence of PAHs sources, namely domestic heating and heavier road traffic (Ratola et al. 2010). Moreover, in colder months, increased partitioning may enhance the accumulation of PAHs in pine needles, whereas in warmer months, increased volatilization and photodegradation may cause losses in the PAHs load of the needle (Amigo et al. 2011; Ratola et al. 2010). Same patterns can be observed also for broadleaved trees (De Nicola et al. 2005). According to Ratola et al. (2010), proportion of heavy compounds in the total amount of PAHs is higher during the warmer seasons. This phenomenon may be caused simply by better weather conditions (less wind and rain), that promote preservation of heavy PAHs deposited on the leaf surface, combined with higher volatilization rates in case of light PAHs. Moreover, there is higher potential influx of heavy PAHs in warmer seasons (e.g., forest or meadow fires), while the supply of lighter PAHs is enhanced in colder periods (e.g., traffic or heating). However, during our research, observed concentrations of heavy PAHs were stable over time, with slight increases in colder periods. It might have been caused by the use of charcoal and wood for residential heating, especially in case of areas surrounding PB3.

## Conclusions

As far as PAHs profiles are concerned, only PB3 located in the Bieszczady National Park, differed significantly from other bogs with the concentrations of pyrogenic PAHs. This difference resulted in the highest carcinogenic potential observed in this location. Emission sources, that produce high amounts of heavy PAHs in the Bieszczady National Park, are traditional kilns for charcoal production.

All studied species had the same qualitative characteristics of accumulated PAHs and differ only in their concentrations. Scots pine was characterized by the lowest accumulation potential from the studied species. The highest accumulation potential was observed for wild rosemary leaves which accumulated the highest amounts of both light and heavy PAHs. Therefore, they were characterized by the highest carcinogenic potential. This finding seems to be of a considerable importance; hence, people living in the forested areas gather wild rosemary shoots and keep them at home for their fragrance and insecticide properties.

Seasonal changes in PAHs concentrations followed the pattern of winter increase, caused mainly by heating season, and summer decrease, caused mainly by volatilization of light PAHs.

## Electronic supplementary material

ESM 1 (DOC 228 kb)
