# Supplementary material for: Interspecific differences in foliar 1 PAHs load between Scots pine, birch, and wild rosemary from three polish peat bogs
Source: Environ Monit Assess. 2016 Jul 8;188(8):456. doi: 10.1007/s10661-016-5465-2 (PMC4938853; doi:10.1007/s10661-016-5465-2)
Supplement: Supplementary file 1 — (DOC 228 kb) [file 10661_2016_5465_MOESM1_ESM.doc]

APPENDIX

Tab.1. Chemical composition of standard reference material used as a quantification standard during gas chromatography–low resolution mass spectrometry analysis.

| Compounds PAHs | Selected ions for mass spectrometry quantification | | R ^2^ in calibration | Mean recovery (%) |
| --- | --- | --- | --- | --- |
|  |  |  |  |  |
|  | Range | *m/z* |  |  |
| acenaphthene | 151–153 | 152 | 0.99 | 67.15 |
|  |  |  |  |  |
| acenaphthylene | 152–154 | 153 | 0.99 | 80.54 |
|  |  |  |  |  |
| fluorene | 165–167 | 166 | 0.99 | 58.13 |
|  |  |  |  |  |
| phenanthrene | 177–179 | 178 | 0.98 | 102.56 |
|  |  |  |  |  |
| anthracene | 177–179 | 178 | 0.98 | 96.66 |
|  |  |  |  |  |
| fluoranthene | 201–203 | 202 | 0.99 | 77.51 |
|  |  |  |  |  |
| pyrene | 201–203 | 202 | 0.99 | 83.02 |
|  |  |  |  |  |
| benzo[a]anthracene | 227–229 | 228 | 0.97 | 78.16 |
|  |  |  |  |  |
| chrysene | 227–229 | 228 | 0.96 | 66.99 |
|  |  |  |  |  |
| benzo[b]fluoranthene | 251–253 | 252 | 0.96 | 101.45 |
|  |  |  |  |  |
| benzo[k]fluoranthene | 251–253 | 252 | 0.98 | 120.55 |
|  |  |  |  |  |
| benzo[a]pyrene | 251–253 | 252 | 0.98 | 68.55 |
|  |  |  |  |  |
| indeno[123-cd]pyrene | 275–277 | 276 | 0.98 | 79.28 |
| dibenzo[ah]anthracene | 275–277 | 276 | 0.98 | 133.44 |
|  |  |  |  |  |
|  |  |  |  |  |
| benzo[ghi]perylene | 277–279 | 278 | 0.98 | 49.81 |

Tab.2. Comparison of concentrations of 16 PAHs in plant samples from three studied peat bogs (* p values < 0.1, ** p values < 0.05, *** p values < 0.001).

| PAHs concentrations [µg/g] | | PB1 (N=12) | PB2 (N=12) | PB3 (N=12) | Friedman ANOVA p value |
| --- | --- | --- | --- | --- | --- |
| acenaphthene | mean | 2.6 | 4.7 | 3.9 | 0.0153** |
|  | median | 2.5 | 4.0 | 4.0 |  |
|  | range | 0.0-4.0 | 2.0-10.0 | 2.0-9.0 |  |
| acenaphthylene | mean | 20.8 | 39.2 | 23.2 | 0.2636 |
|  | median | 15.5 | 17.0 | 21.0 |  |
|  | range | 3.0-94.0 | 7.0-171.0 | 11.0-49.0 |  |
| fluorene | mean | 33.1 | 42.3 | 36.3 | 0.4474 |
|  | median | 27.5 | 34.0 | 28.0 |  |
|  | range | 11.0-67.0 | 5.0-117.0 | 9.0-111.0 |  |
| phenanthrene | mean | 356.9 | 394.3 | 326.6 | 0.1738 |
|  | median | 291.0 | 369.5 | 281.5 |  |
|  | range | 62.0-911.0 | 16.0-849.0 | 4.0-1207.0 |  |
| anthracene | mean | 6.4 | 10.8 | 9.0 | 0.1205 |
|  | median | 4.5 | 10.5 | 6.5 |  |
|  | range | 2.0-18.0 | 1.0-21.0 | 1.0-22.0 |  |
| fluoranthene | mean | 65.7 | 104.7 | 96.1 | 0.0671* |
|  | median | 62.0 | 100.5 | 89.5 |  |
|  | range | 0.0-156.0 | 0.0-249.0 | 2.0-207.0 |  |
| pyrene | mean | 29.5 | 50.7 | 55.2 | 0.1738 |
|  | median | 31.0 | 42.0 | 56.5 |  |
|  | range | 4.0-61.0 | 0.0-114.0 | 1.0-110.0 |  |
| benzo[a]anthracene | mean | 3.4 | 2.3 | 12.1 | 0.1371 |
|  | median | 3.0 | 5.5 | 12.5 |  |
|  | range | 0.0-9.0 | 0.0-13.0 | 0.0-27.0 |  |
| chrysene | mean | 10.2 | 19.0 | 32.3 | 0.0036** |
|  | median | 10.0 | 19.5 | 39.5 |  |
|  | range | 0.0-21.0 | 0.0-47.0 | 0.0-63.0 |  |
| benzo[b]fluoranthene | mean | 4.8 | 7.0 | 20.9 | 0.0617* |
|  | median | 4.0 | 6.5 | 23.5 |  |
|  | range | 0.0-12.0 | 0.0-14.0 | 0.0-47.0 |  |
| benzo[k]fluoranthene | mean | 1.9 | 2.5 | 11.6 | 0.0015** |
|  | median | 0.0 | 1.5 | 11.5 |  |
|  | range | 0.0-8.0 | 0.0-8.0 | 0.0-29.0 |  |
| benzo[e]pyrene | mean | 2.7 | 6.9 | 15.0 | 0.0161** |
|  | median | 1.5 | 5.0 | 15.5 |  |
|  | range | 0.0-9.0 | 0.0-36.0 | 0.0-35.0 |  |
| benzo[a]pyrene | mean | 2.7 | 3.1 | 16.9 | 0.0423** |
|  | median | 1.5 | 4.0 | 15.5 |  |
|  | range | 0.0-9.0 | 0.0-8.0 | 0.0-42.0 |  |
| dibenzo[ah]anthracene | mean | 0.0 | 0.9 | 0.4 | 0.6065 |
|  | median | 0.0 | 0.0 | 0.0 |  |
|  | range | 0.0 | 0.0-11.0 | 0.0-5.0 |  |
| indeno[123-cd]pyrene | mean | 0.5 | 1.3 | 8.9 | 0.0076** |
|  | median | 0.0 | 0.0 | 10.0 |  |
|  | range | 0.0-6.0 | 0.0-9.0 | 0.0-32.0 |  |
| benzo[ghi]perylene | mean | 0.5 | 1.1 | 11.3 | 0.0014** |
|  | median | 0.0 | 0.0 | 10.5 |  |
|  | range | 0.0-6.0 | 0.0-8.0 | 0.0-34.0 |  |

Tab.3. Comparison of percentage participation of 3, 4 and 5 plus 6 ring PAHs in total PAHs in samples from three studied peat bogs (* p values < 0.1, ** p values < 0.05, *** p values < 0.001).

| [%] of total PAHs | | PB1 (N=12) | PB2 (N=12) | PB3 (N=12) | Friedman ANOVA p value |
| --- | --- | --- | --- | --- | --- |
| 3R | mean | 77.8 | 73.3 | 63.7 | 0.0065** |
|  | median | 77.4 | 67.2 | 57.7 |  |
|  | range | 66.1-91.7 | 58.2-100.0 | 43.6-96.0 |  |
| 4R | mean | 20.0 | 23.8 | 25.7 | 0.0438* |
|  | median | 21.1 | 26.6 | 29.0 |  |
|  | range | 8.3-31.6 | 0.0-39.1 | 4.0-36.6 |  |
| 5+6R | mean | 2.2 | 2.9 | 10.7 | 0.0757 |
|  | median | 1.8 | 2.6 | 11.7 |  |
|  | range | 0.0-7.7 | 0.0-8.6 | 0.0-22.1 |  |

Tab.4. Comparison of concentrations of 16 PAHs in samples of *P.sylvestris*, *Betula* spp. and *R.tomentosum* (* p values < 0.1, ** p values < 0.05, *** p values < 0.001).

| PAHs concentrations [µg/g] | | *P.sylvestris* (N=12) | *Betula* spp. (N=12) | *R.tomentosum* (N=12) | Friedman ANOVA p value |
| --- | --- | --- | --- | --- | --- |
| acenaphthene | mean | 4.4 | 2.7 | 4.1 | 0.0226** |
|  | median | 4.0 | 2.5 | 4.0 |  |
|  | range | 0.0-10.0 | 2.0-4.0 | 2.0-6.0 |  |
| acenaphthylene | mean | 27.8 | 22.3 | 33.1 | 0.5875 |
|  | median | 16.0 | 15.5 | 21.0 |  |
|  | range | 3.0-142.0 | 8.0-94.0 | 12.0-171.0 |  |
| fluorene | mean | 35.0 | 29.0 | 47.8 | 0.0590* |
|  | median | 25.5 | 28.0 | 38.5 |  |
|  | range | 5.0-111.0 | 18.0-67.0 | 28.0-117.0 |  |
| phenanthrene | mean | 147.9 | 380.6 | 549.3 | 0.0003*** |
|  | median | 159.0 | 351.0 | 427.0 |  |
|  | range | 4.0-424.0 | 175.0-729.0 | 242.0-1207.0 |  |
| anthracene | mean | 4.3 | 9.9 | 12.0 | 0.0013** |
|  | median | 4.0 | 7.5 | 11.0 |  |
|  | range | 1.0-11.0 | 2.0-20.0 | 2.0-22.0 |  |
| fluoranthene | mean | 37.8 | 112.8 | 115.8 | 0.0001*** |
|  | median | 20.0 | 93.0 | 101.5 |  |
|  | range | 0.0-119.0 | 36.0-249.0 | 60.0-207.0 |  |
| pyrene | mean | 27.7 | 50.0 | 57.8 | 0.0263** |
|  | median | 15.5 | 41.5 | 46.5 |  |
|  | range | 0.0-114.0 | 18.0-105.0 | 29.0-110.0 |  |
| benzo[a]anthracene | mean | 4.3 | 5.8 | 10.7 | 0.0254** |
|  | median | 2.0 | 4.0 | 8.0 |  |
|  | range | 0.0-20.0 | 2.0-14.0 | 3.0-27.0 |  |
| chrysene | mean | 10.8 | 23.4 | 27.4 | 0.0099** |
|  | median | 7.0 | 15.5 | 20.5 |  |
|  | range | 0.0-41.0 | 9.0-63.0 | 9.0-61.0 |  |
| benzo[b]fluoranthene | mean | 6.4 | 7.9 | 18.4 | 0.0070** |
|  | median | 3.5 | 5.0 | 11.5 |  |
|  | range | 0.0-35.0 | 0.0-31.0 | 3.0-47.0 |  |
| benzo[k]fluoranthene | mean | 2.8 | 2.4 | 10.8 | 0.0001*** |
|  | median | 0.0 | 0.0 | 7.0 |  |
|  | range | 0.0-19.0 | 0.0-15.0 | 0.0-29.0 |  |
| benzo[e]pyrene | mean | 3.7 | 8.7 | 12.3 | 0.0018** |
|  | median | 0.0 | 5.0 | 7.5 |  |
|  | range | 0.0-24.0 | 0.0-36.0 | 0.0-35.0 |  |
| benzo[a]pyrene | mean | 3.8 | 5.8 | 13.1 | 0.0053** |
|  | median | 0.0 | 5.0 | 6.0 |  |
|  | range | 0.0-29.0 | 0.0-18.0 | 0.0-42.0 |  |
| dibenzo[ah]anthracene | mean | 0.0 | 0.0 | 1.3 | 0.1353 |
|  | median | 0.0 | 0.0 | 0.0 |  |
|  | range | 0.0 | 0.0 | 0.0-11.0 |  |
| indeno[123-cd]pyrene | mean | 2.5 | 2.3 | 5.8 | 0.0863* |
|  | median | 0.0 | 0.0 | 0.0 |  |
|  | range | 0.0-19.0 | 0.0-12.0 | 0.0-32.0 |  |
| benzo[ghi]perylene | mean | 2.8 | 2.2 | 7.9 | 0.0056** |
|  | median | 0.0 | 0.0 | 2.5 |  |
|  | range | 0.0-23.0 | 0.0-15.0 | 0.0-34.0 |  |
